# Basic clinical retroperitoneal anatomy for pelvic surgeons

**DOI:** 10.4274/tjod.88614

**Published:** 2019-01-09

**Authors:** İlker Selçuk, Burak Ersak, İlkan Tatar, Tayfun Güngör, Emre Huri

**Affiliations:** 1 University of Health Sciences, Ankara Dr. Zekai Tahir Burak Woman’s Health Training and Research Hospital, Clinic of Gynecologic Oncology, Ankara, Turkey; 2 Hacettepe University Faculty of Medicine, Department of Anatomy, Ankara, Turkey; 3 Hacettepe University Faculty of Medicine, Department of Urology, Ankara, Turkey

**Keywords:** Surgery, anatomy, hypogastric, ureter, gynecology

## Abstract

Basic anatomical knowledge should be improved during residency period with clinical practice. Especially pelvic surgeons; obstetricians, gynecologists, gynecological oncologists, urologists and general surgeons must have an advanced level practise of retroperitoneal anatomy to gain surgical skills. Retroperitoneal topographic anatomy, retroperitoneal vasculature, ureteric dissection and pelvic avascular spaces are the precise points during pelvic surgery.

## Retroperitoneum

## 1. Topographic retroperitoneal anatomy

The posterior abdominal wall is the posterior boundary of the abdominal cavity, which is the continuous part of posterior thoracic wall from the level of diaphragm cranially and posterior pelvic wall caudally. The lumbar vertebra, pelvic girdle, posterior abdominal wall muscles [musculus (m) quadratus lumborum, m. psoas major, m. psoas minor, m. iliacus, and muscles of diaphragm] and their fascia are the members of this region.

**Figure 1:**
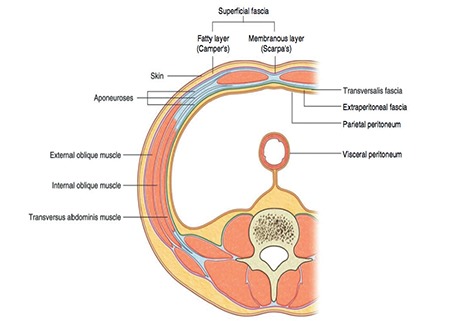
Transverse section of the anterior abdominal wall; the extraperitoneal fascia with fatty tissue under the parietal peritoneum lies on the posterior abdominal wall called the retroperitoneum (Gray’s Anatomy for Students, 3^rd^ Edition, Churchill Livingstone/Elsevier, 2015)^(1)^

**Figure 2:**
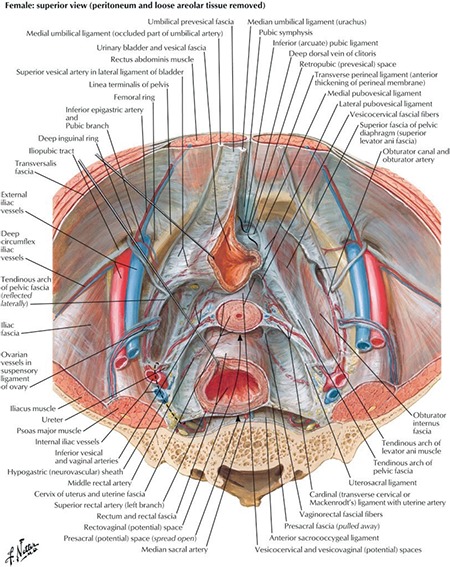
Pelvic viscera and retroperitoneum (Atlas of Human Anatomy, 6^th^ Edition, Saunders/Elsevier, 2014)^(2)^

The retroperitoneum is a part of the abdominal cavity; surrounded anteriorly by the parietal peritoneum and posteriorly by the transversalis fascia (Figure 1)^(1)^. It is a wide area from the pelvis to the diaphragm and contains numerous organs and structures; structures behind the peritoneum are called ‘retroperitoneal’ (Figure 2)^(2)^. The primary retroperitoneal organs are the adrenal glands, kidneys, ureter, the abdominal aorta, inferior vena cava and their branches. The secondary retroperitoneal organs, which were initially intraperitoneal and became retroperitoneal structures during embryologic development due to the regression of peritoneal tissue lying on the posterior wall of the abdominal cavity (the mesentery of these structures fuse with the posterior abdominal wall), are the ascending and descending colon, duodenum except the bulbus part (first half of duodenum segment 1) and pancreas.

### Clinical tip: How to enter the retroperitoneal area?

When there is a distorted anatomy of peritoneal structures, to achieve a normal anatomy and resect all pathologic lesions, the surgeon needs to gain access to the retroperitoneum, which is mainly safe and that leads to a comprehensive dissection and visualization of the relevant anatomy.

**Figure 3:**
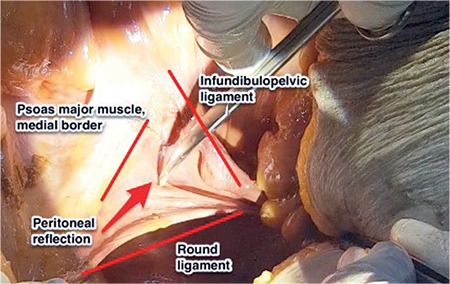
Demonstration to enter the retroperitoneum between the round ligament and infundibulopelvic ligament (lateral parietal peritoneum), right pelvic side wall (cadaveric dissection)

**Figure 4:**
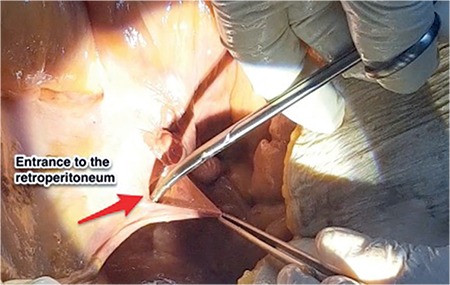
Demonstration of opening retroperitoneum, right pelvic side wall (cadaveric dissection)

The peritoneal reflection between the round ligament [ligamentum (lig) teres uteri] (lateral) and infundibulopelvic ligament (medial) is an easy way to enter the retroperitoneum (Figure 3). Gentle traction on the lateral parietal peritoneal surface and cutting it either with scissors or energy devices is an easy and safe way of opening the retroperitoneum (Figure 4).

## 2. Pelvic retroperitoneal vasculature

### A. Arteries

### Abdominal aorta

**Figure 5:**
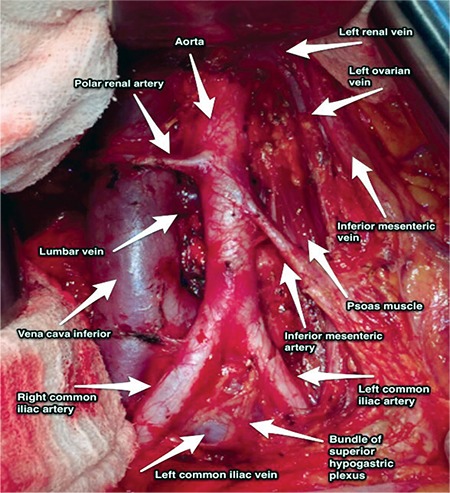
Paraaortic region, aorta and inferior vena cava after paraaortic lymphadenectomy (surgical archieve)

The thoracic aorta is called the abdominal aorta because it enters the abdominal cavity through the diaphragm and it lies at the posterior abdominal wall, anterior to the vertebral column. The abdominal aorta is divided into the right and left common iliac arteries at the level of the L4-L5 vertebra, and the common iliac artery is divided into two parts as the external and internal iliac artery at the pelvic brim. The ovarian artery, median sacral artery, external iliac artery, internal iliac artery and its branches are important structures of pelvic retroperitoneal vasculature (Figure 5).

**Ovarian artery:** This is located on the anterolateral surface of the abdominal aorta, at the level of the L2 vertebra, generally 2 cm below the level of the left renal vein. On the left side, it goes over the psoas major muscle and enters the pelvic cavity by crossing the common iliac artery. On the right side, first it crosses over the anterior surface of inferior vena cava then goes downward beside the ascending colon 1 cm above the right ureter and enters the pelvic cavity by crossing over the common iliac artery or sometimes the external iliac artery.

**Median sacral artery:** This is the continuation of the abdominal aorta on the anterior surface of the sacrum and coccyx. It is crossed by the left common iliac vein and care should be taken during hysteropexy and colpopexy operations.

**Figure 6:**
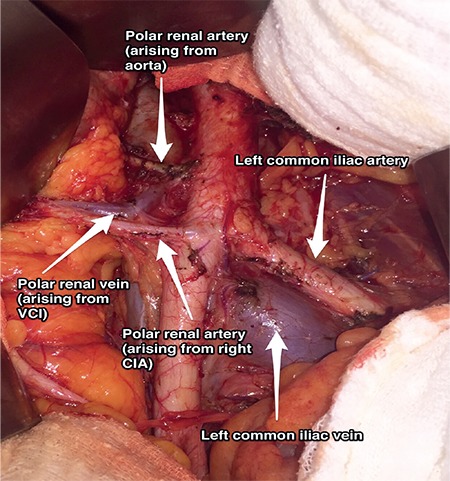
Polar renal artery arising from the right common iliac artery and also abdominal aorta (surgical archive) VCI: Vena cava inferior, CIA: Common iliac artery

**Common iliac artery: **The common iliac artery divides into the external and internal iliac artery. It is the point where polar renal arteries mostly arise (Figure 6); meticulous dissection is important during surgical procedures regarding this field^(3)^.

**Figure 7:**
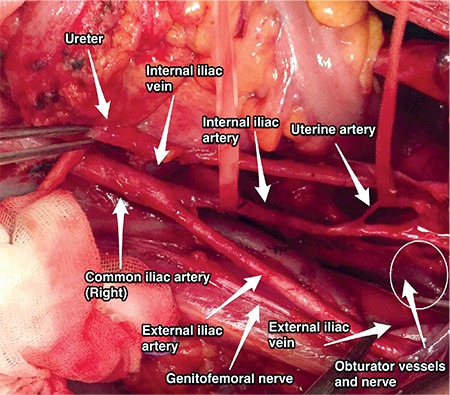
Uterine artery, right pelvic side wall (surgical archive)

**External iliac artery: **This goes along the medial border of the psoas muscle to the level of femoral ring, which is below the inguinal ligament. The genitofemoral nerve is found on the lateral border of the external iliac artery (lateral border of pelvic lymphadenectomy) (Figure 7). It is the principal artery of the lower limb. Its branches are the deep circumflex femoral artery and the inferior epigastric artery.

**Internal iliac artery: **This runs infero-medially after the pelvic brim and is the major vascular supply of the pelvic cavity. It has two trunks; posterior and anterior^(4)^. The branches of the posterior trunk are the superior gluteal artery, lateral sacral artery, and iliolumbar artery. The branches of the anterior trunk are the umbilical, uterine, superior and inferior vesical, vaginal, obturator, middle rectal, internal pudendal and inferior gluteal artery (Figure 7).

### Clinical tip: Internal iliac artery and peripartum bleeding

During intractable pelvic hemorrhage or peripartum bleeding, ligation of the anterior trunk of the internal iliac artery bilaterally will decrease the amount of bleeding dramatically because the internal iliac artery is the major vascular supply of the pelvic cavity.

**Umbilical artery:** This is the end artery of the internal iliac artery (anterior trunk). It goes longitudinally to the abdominal wall and becomes the medial umbilical ligament. When traction is applied to the umbilical artery during laparoscopic procedures, it will indicate the origin of the uterine artery.

**Uterine artery: **The uterine artery arises from the anterior trunk and goes medially through the broad ligament (lig. latum uteri) [within the cardinal ligament (lig. transversum cervicis)] towards the isthmic portion of the uterus to supply the uterus and cervix (Figure 7). It crosses the ureter close to the uterus.

### B. Veins

### Inferior vena cava

The inferior vena cava (IVC) begins just inferior to the L5 vertebra, where the abdominal aorta has a bifurcation of common iliac arteries. Under the level of the umbilicus, it is slightly at the posterior plane of the abdominal aorta. It ascends over the right psoas major muscle, right to the aorta, and above the level of umbilicus it gets closer to the anterior line of the abdominal aorta (Figure 5).

### Clinical tip: Renal vein and ovarian vein

The left renal vein crosses over the abdominal aorta below the origin of the superior mesenteric artery and drains into the vena cava inferior. It receives blood from the left ovarian and adrenal veins and ascending lumbar vein. On the right side, the ovarian vein enters directly into the IVC.

**External iliac vein: **This is the continuation of the femoral vein above the inguinal ligament and runs on the posterior side of the external iliac artery.

**Figure 8:**
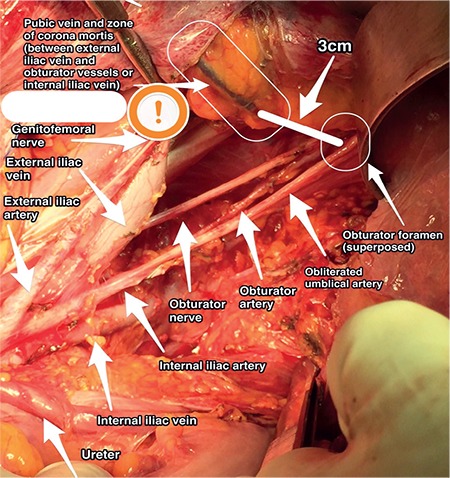
Pubic vein, left pelvic side wall (surgical archive)

**Pubic vein: **This is a vascular connection between the external iliac/inferior epigastric and obturator vein, and hemorrhage of this vein is called corona mortis. It is on the posterior part of pubic bone over the obturator fossa (Figure 8). This area is dissected during pelvic lymphadenectomy in gynecologic oncology practice, and the surgeon should be careful to prevent hemorrhage from this venous connection^(5).^

**Internal iliac vein: **Corresponding branches of the internal iliac artery generally run with their veins. There are numerous anomalous and collateral veins that drain into the internal iliac vein.

**Common iliac vein: **This starts from the conjunction point of the internal and external iliac veins and forms the inferior vena cava with its counterpart.

### Clinical tip: Promontorium

It is at the upper part of pelvic cavity on the medial side of sigmoid colon. Transparietal fixation of the perisigmoid and perirectal fatty tissue or fixation of the sigmoid colon by appendix epiploicas after mobilization may provide adequate exposure of this field. The median sacral artery and left common iliac vein are just superior to the promontorium and the internal iliac artery with the ureter are in close connection at the lateral part.

### Clinical tip: Left common iliac vein

**Figure 9:**
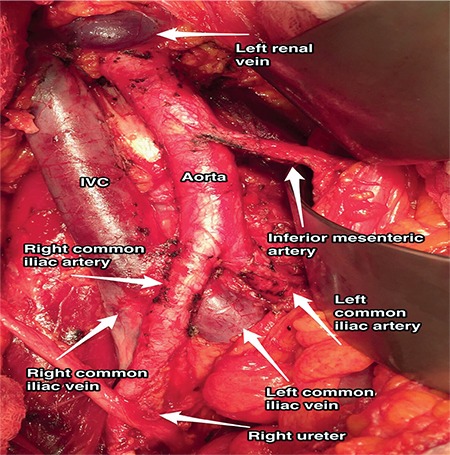
Left and right common iliac veins and arteries (surgical archive) IVC: Inferior vena cava

It is a potential danger point during dissection of the field of the promontorium, which lies on the medial part of the left common iliac artery (Figure 9). During laparoscopic surgery, obesity and bad trocar angles will increase the likelihood of an injury to the left common iliac vein.

## 3. Ureter

The ureter is a muscular structure, functioning in the transport of urine from the kidney to the bladder. It is about 23-30 cm in length. The renal pelvis narrows as it passes through the hilum of the kidney and forms the ureter, which continues inferiorly. After crossing the bifurcation of the common iliac arteries or the origin of the external iliac artery over the pelvic brim, it goes on the medial side of the psoas major muscle and runs along the posterior leaf of the broad ligament before entering the urinary bladder. 

The distal ureter is crossed by the uterine artery antero-superiorly. The end part of the distal ureter enters the bladder obliquely to the smooth muscle wall of the bladder, providing a sphincter-like action.

### The narrowest points of the ureter:

-The ureteropelvic junction

-Pelvic brim, where the ureters cross the common iliac vessels

-The ureterovesical junction, where the ureters enter the smooth muscle wall of the bladder

**Figure 10:**
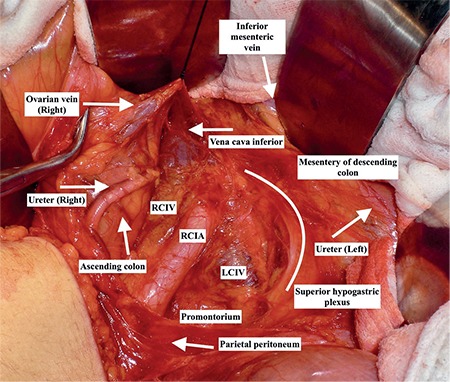
Right ureter below the right ovarian vein medial to the ascending colon and lateral to inferior vena cava, and left ureter underneath the mesentery of descending colon, medial/ parallel to the inferior mesenteric vein and lateral to aorta/ superior hypogastric plexus (surgical archive) RCIV: Right common iliac vein, RCIA: Right common iliac artery, LCIV: Left common iliac vein

Anatomically, the ureter is divided into abdominal, pelvic, and intravesical parts. The abdominal part is on the medial border of the psoas muscle fascia over the genitofemoral nerve. The right ureter starts from the level where the posterior of the second part of duodenum is found and descends within the peritoneum of the ascending colon close to the right colic and ileocolic artery, lateral to the root of the small bowel mesentery and inferior vena cava under the ovarian vessels. Afterwards, it passes posterior to the terminal ileum and cecum. The left ureter descends on the lateral part of the abdominal aorta over the psoas muscle fascia and crossed anteriorly by the left colic artery and ovarian vessels. During this course of the left ureter, it lies parallel to the inferior mesenteric vein (Figure 10) and passes along the posterior of the sigmoid colon. The ureters cross the bifurcation of the common iliac artery over the pelvic brim. 

When the ureter enters the true pelvis, it runs inferior to the ovarian vessels, and goes through that path to the bladder on the posterior leaf of the broad ligament. It goes antero-medially while crossing the uterine artery (water under the bridge) afterwards, it enters the ureteric tunnel (web tunnel) within the cardinal ligament. The ureter passes lateral to the antero-lateral vaginal fornix within the bladder pillar and enters the trigone of the bladder. The orifices of the ureters are seen on the postero-lateral part of the trigone. The ureters take a 1.5-2 cm course in the bladder wall.

**Figure 11:**
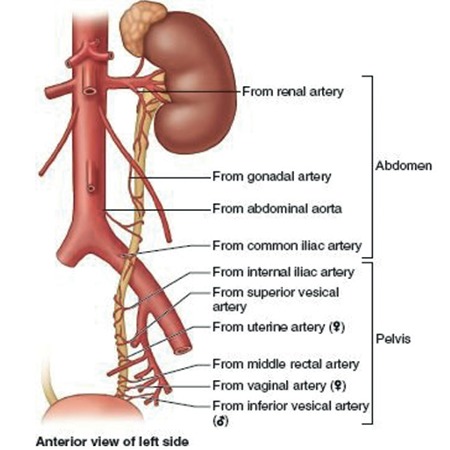
Vascularization of ureter from the kidney to the bladder (left side), while dissecting the ureter traction should be applied towards the side of blood vessels (Moore Clinically Oriented Anatomy, 7^th^ Edition, Wolters Kluwer/Lippincott Williams & Wilkins, 2013)^(6)^

The ureter does not have a primary arterial vessel for blood supply, it receives arterial branches from the renal, ovarian, common iliac, internal iliac, uterine, superior gluteal, vaginal, middle rectal, inferior and superior vesical arteries through its pathway from the renal pelvis to the bladder (Figure 11)^(6)^.

These blood vessels anastomose with each other and shape a continuous longitudinal blood supply. 

The ureters are very rich in innervation and they shape the ureteric plexus. The primary sensation of the ureter (visceral afferent fibers) is provided by nerves from T12-L2 (sympathetic system). Visceral efferent fibers come from both sympathetic and parasympathetic bundles.

### Clinical tip: Vascularization of ureter

**Figure 12:**
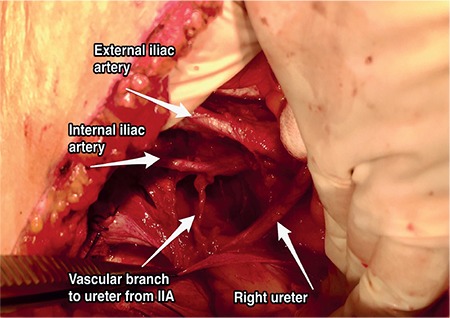
Vascular branch to ureter from internal iliac artery, right pelvic side wall, in the pelvis the most important vascular supply of the ureter is the branch from the internal iliac artery (surgical archive) IIA: Internal iliac artery

Injury to the longitudinal blood vessels of the ureter may cause ischemia or necrosis on the adventitia of ureter. If the adventitia of the ureter is not stripped or the fatty tissue over it (mesoureter in clinical term) has not been sacrificed, the surgical mobilisation of the ureter could easily be performed while avoiding injuries. Internal iliac artery is the most important vascular supply of ureter in the pelvis (Figure 12).

### Clinical tip: Ureter injuries

During infundibulopelvic (IP) ligament ligation, where the ureter passes inferior to it, especially when the anatomy is distorted because of tumors, masses or severe adhesions, the IP ligament should be isolated and the ureter must be dissected to avoid injuries.

The ureter, where it crosses under the uterine artery above the vaginal artery, near the isthmic part of the uterus, is a site of injury during uterine artery ligation while performing hysterectomy. The ureter stands very close to the cervix, and to avoid injuries, the uterus must be pulled towards the other side cranially to maximize the distance between the ureter and the cervix. 

**Figure 13:**
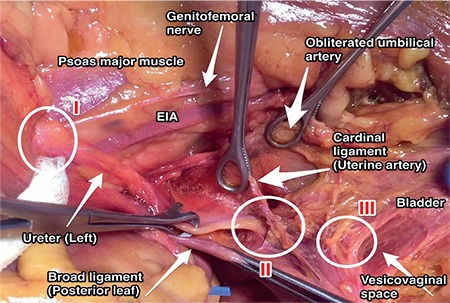
Sites of ureter injury, left pelvic side wall: Zone I, during infundibulopelvic ligament ligation just below the level of pelvic inlet; zone II, during uterine artery ligation (ureter crosses the cardinal ligament-uterine artery complex); zone III, during vaginal excision (ureter is anterolateral to the anterior vagina before entering the bladder-trigone) (cadaveric dissection) EIA: External iliac artery

After crossing the uterine artery, the ureter passes very close to the anterolateral part of vagina and during cardinal-uterosacral ligament ligation, the ureter will be injured (Figure 13).

## 4. Avascular spaces in the pelvis

**Figure 14:**
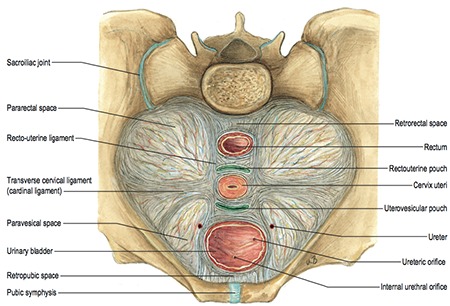
Avascular spaces and supporting ligaments in the pelvis (Sobotta Atlas of Human Anatomy, 15^th^ Edition, Elsevier, Urban&Fischer. Copyright 2013/Gray’s Anatomy, The Anatomical Basis of Clinical Practice, 41^th^ edition, Elsevier, 2016)^(7)^

Pelvic connective tissue divides the subperitoneal pelvic area into different spaces. These spaces are filled by fatty or loose areolar connective tissues, which are generally avascular. These potential spaces have a role in the functioning of urinary, reproductive, and gastrointestinal systems. They have a crucial role in the management of pelvic operations because knowing them exactly allows restoration of normal anatomy and avoids injury of pelvic viscera and structures. These pelvic spaces are as follows (Figure 14)^(7)^:

- Retropubic (Retzius) space

- Paravesical space

- Presacral (Retrorectal) space

- Pararectal space

- Vesicovaginal (Vesicouterine) space

- Rectovaginal space

### Retropubic (Prevesical/Retzius) space

**Figure 15:**
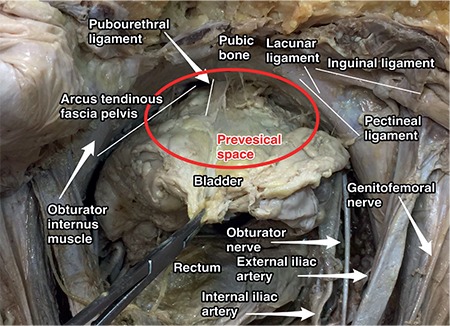
Prevesical space and contents (cadaveric dissection)

This is the potential extraperitoneal space between the bladder and the pubic bone that generally contains fat. Its boundaries are (Figure 15):

- Anteriorly: Pubic symphysis,

- Posteriorly: Bladder,

- Superiorly: Parietal peritoneum (anterior abdominal wall),

- Laterally: Arcus tendinous fascia pelvis and ischial spines.

Grasping the median umbilical ligament (Urachus) with downward traction and cutting it will open the space of Retzius. The dorsal clitoral neurovascular bundle is found at the midline, and the obturator nerve bundle is located on the lateral plane. An accessory obturator artery from the external iliac artery that runs along the posterior part of pubic bone or a pubic vein from the external iliac vein or an arterial branch from the inferior epigastric artery will be detected at the lateral border of that field during its path to obturator foramen. Moreover, lateral to the bladder neck and urethra, nerves innervating the bladder and urethra and a venous plexus (Santorini plexus), (which could be injured during suture placement for Burch retropubic colposuspension) are found.

### Clinical tip: Burch colposuspension

This is a retropubic colposuspension operation for stress urinary incontinence in which the sutures starting from the paravaginal tissue are anchored to the ileopectineal ligament (Cooper’s ligament), the superior border of the ischiopubic rami to maintain the tension on bladder neck and urethra.

### Paravesical (and paravaginal) space

**Figure 16:**
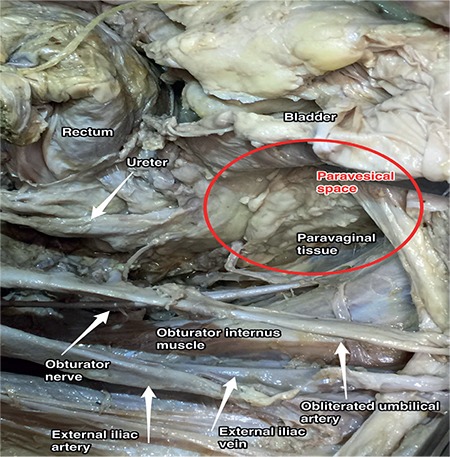
Paravesical space, right pelvic side wall (cadaveric dissection)

This is located within the lateral part of the Retzius space anterior to the cardinal ligament, bilaterally. Its boundaries are (Figure 16):

- Superiorly: Lateral umbilical folds (peritoneal thickening of inferior epigastric vessels),

- Inferiorly: Pubocervical fascia where it enters into the tendinous structure of levator ani muscle, iliococcygeus muscle,

- Anteriorly: Superior pubic ramus, arcuate line of the os ilium,

- Posteriorly: Endopelvic fascial sheath that covers the internal iliac artery and vein, cardinal ligament which separates it from the anterior part of pararectal space and uterine artery,

- Medially: Bladder pillars,

- Laterally: Pelvic side wall, obturator internus and levator ani muscle.

Lateral to the median umbilical ligament, after detecting the lateral border of bladder, the medial umbilical ligament (obliterated umbilical artery) can be identified and it divides the paravesical space into two parts (medial and lateral). The lateral part is the obturator space (under the external iliac vessels) and the medial part is the ventral parametrium. The obturator space contains (Figure 8) the obturator nerve, obturator artery and vein, and fatty and lymphatic tissue. During pelvic lymphadenectomy to dissect the obturator lymph nodes, the surgeon needs to open the paravesical space first then dissect the lateral part on the pelvic side wall. Over the obturator fossa, there are numerous anomalous and collateral vessels, which need tiny and careful dissection to prevent hemorrhage due to injury^(8)^. Paravesical space contains obliterated umbilical artery and it is in close relation with obturator neurovascular bundle and external iliac vessels with the lymphatic and fatty tissue^(9)^.

### Clinical tip: Paravesical space

The paravesical space is generally accessed during Burch colposuspension, paravaginal defect repair, pelvic lymphadenectomy and some endometriosis operations after opening the retroperitoneal space entirely.

Radical hysterectomy is another surgical procedure that needs adequate exposure of the paravesical space during the operation. After opening the retroperitoneal space by transecting the round ligament and cutting the anterior leaf of broad ligament infero-medially, the place lateral to the median umbilical ligament (medial to round ligament), adjacent to the bladder, is the paravesical space and it develops inferiorly to the level of levator ani muscle.

### Presacral (retrorectal) space

**Figure 17:**
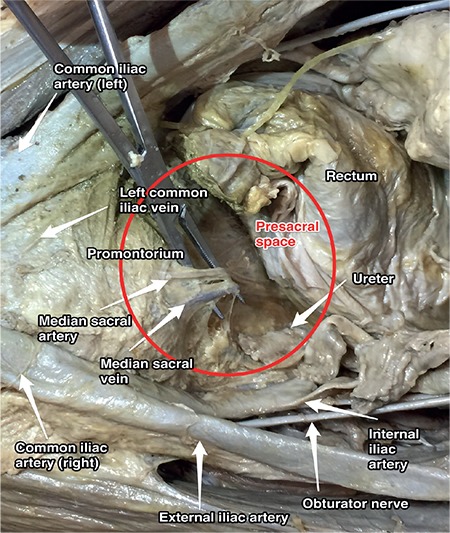
Presacral space (cadaveric dissection)

The retrorectal space is between the rectum and the sacral-coccygeal part of spine. The presacral space is a retroperitoneal area, which is between the presacral fascia of the sacrum (Waldeyer’s fascia) and parietal peritoneum of the posterior abdominal wall. Its boundaries are (Figure 17):

- Superiorly: Peritoneal (parietal) reflections,

- Anteriorly: Distal portion of the sigmoid mesentery, posterior rectal fascia, rectum,

- Posteriorly: Anterior longitudinal ligament, sacral promontorium and anterior part of the sacrum,

- Inferiorly: Levator ani and coccygeus muscle,

- Laterally: Ureter, internal iliac vessels and hypogastric nerves.

The presacral space starts from the parietal peritoneal reflection at the rectosigmoid junction to the pelvic bottom, which contains fatty tissue, lymph nodes, nerve plexuses and blood vessels, median sacral vessels (the artery is from the aorta) and superior rectal vessels (the artery is from the inferior mesenteric artery).

### Clinical tip: Presacral space

**Figure 18:**
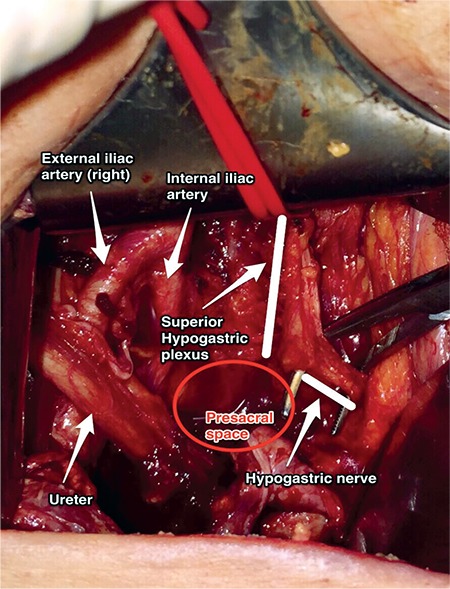
Presacral space and superior hypogastric plexus (surgical archive)

There may be some anatomic variations in this field; presacral anastomoses between the lateral and middle sacral veins need careful dissection during surgery. Moreover, it is very close to the hypogastric nerves and sympathetic trunk. Below the level of the aortic bifurcation, the left common iliac vein crosses the sacral promontorium from right to left. The median sacral artery is detected at the midline or very close to the midline over the sacrum, so care must be taken during sacrocolpopexy procedure and paracoccygeal procedures. The superior hypogastric plexus may also be seen at the superior part of the presacral space over the sacral promontorium (Figure 18).

Primary lesions of this area are rare; however, lesions from adjacent structures may be seen in this field. After rectal or rectosigmoid resections, this field could be a place for accumulation of fluid leakages.

### Clinical tip: Presacral hemorrhage

A serious bleeding may happen because of injuries of the middle sacral artery or vein particularly due to anastomoses. If the vessel structure is retracted into the sacral foramina, it will be harder to control the hemorrhage. A thumb tack could be applied if needed.

### Pararectal space

**Figure 19:**
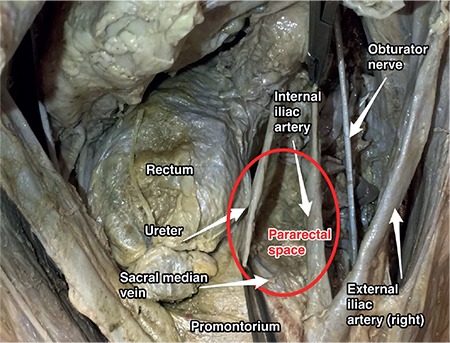
Pararectal space, right pelvic side wall (cadaveric dissection)

The pararectal space is located lateral to the rectum and retrorectal space, and it is at the posterior part of the cardinal ligament. Its boundaries are (Figure 19):

- Anteriorly: Cardinal ligament,

- Medially: Rectal pillars, uterosacral ligament, ureter,

- Laterally: Internal iliac artery,

- Posteriorly: Sacrum,

- Caudally: Puborectalis muscle.

**Figure 20:**
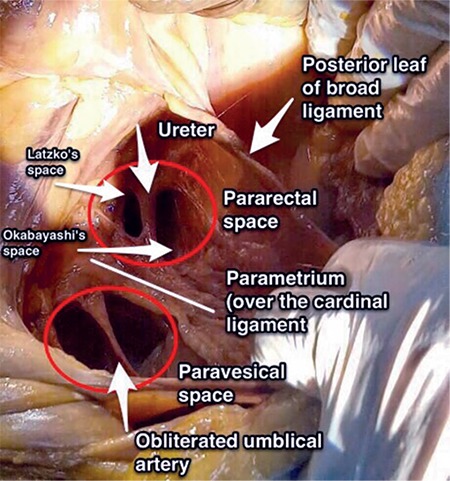
Right pelvic side wall; the paravesical space, anterior to the cardinal ligament is divided into two parts by the obliterated umbilical artery and the pararectal space, posterior to the cardinal ligament is divided into two parts by the ureter, the lateral part is called Latzko’s space and the medial part is called Okabayashi’s space (cadaveric dissection)

It contains fatty and connective tissue, and the ureter passes along the pararectal space at the medial part. After a tiny dissection of the ureter within the pararectal space, the ureter will divide it into two parts; medial is Okabayashi’s space and lateral is Latzko’s space (Figure 20). It is separated from the paravesical space by the cardinal ligament/uterine artery and from the presacral space by the rectal septa.

### Okabayashi’s space

The medial pararectal space is called Okabayashi’s space and it is between the ureter and the rectouterine ligament, which is developed after opening the posterior leaf of the broad ligament (Figure 20).

### Latzko’s space

The lateral pararectal space is called Latzko’s space and it is between the ureter and pelvic side wall, which is developed after dissection of internal iliac artery (Figure 20). Middle rectal artery could be seen at the lateral rectal wall by the way the pelvic splanchnic nerves and the fibers of the inferior hypogastric plexus, which lies under the middle rectal artery, could be preserved.

### Clinical tip: Pararectal space

When the pouch of Douglas (rectovaginal space) is obliterated by a tumor, severe adhesions or endometriosis, the surgeon should primarily open the retroperitoneal space, find the ureter, and after ureterolysis the pararectal space should be developed to dissect the rectum from the vagina and open the rectovaginal space. 

During radical hysterectomy, the pararectal space should be developed to excise the cardinal ligament entirely. After dissection of the posterior leaf of the broad ligament postero-medially, the pararectal space is developed between the ureter and internal iliac artery anterior to the sacrum.

### Vesicovaginal (Vesicouterine) space

**Figure 21:**
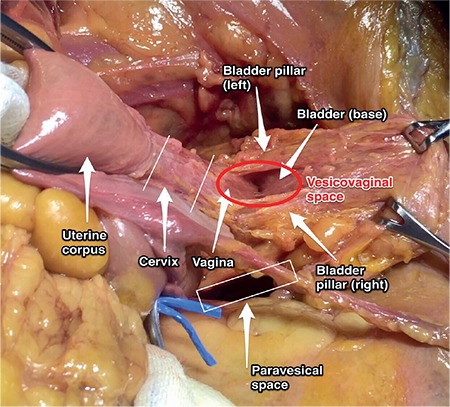
Vesicovaginal space (cadaveric dissection)

The vesicovaginal, vesicocervical, and vesicouterine spaces are all at the same longitudinal axis and this area is also known as the anterior cul-de-sac. The boundaries of the vesicocervical space are (Figure 21):

- Anteriorly: Posterior part of the bladder, 

- Posteriorly: Cervix,

- Laterally: Bladder pillars (superior portion, that is divided by the ureter), pubocervical ligament (vesicouterine ligament in clinical term)^(10)^,

- Superiorly: Anterior peritoneal fold, vesicouterine peritoneal fold.

### The boundaries of the vesicovaginal space:

- Anteriorly: Trigone of bladder,

- Posteriorly: Vagina,

- Laterally: Bladder pillars, pubocervical ligament,

- Inferiorly: Urogenital diaphragm.

After cutting the vesicouterine pouch (between the dome of the bladder and the anterior part of the uterus) and with posterior traction of uterus towards the promontorium, this field can be opened easily. The lateral bladder pillars contain blood vessels, vesical veins (inferior, superior), cervical terminal branches from the uterine artery, and connective tissue from the cardinal ligament.

### Clinical tip: Vesicovaginal space

During radical hysterectomy before excising the anterior parametrium, the bladder should be dissected to the level of the trigone, afterwards the ureter should be dissected from the parametrium. Moreover, vesicovaginal space dissection should be performed medially at the midline within the loose areolar tissue because lateral extensions during dissection could cause bleeding from the bladder pillars (vesical veins).

### Rectovaginal space

**Figure 22:**
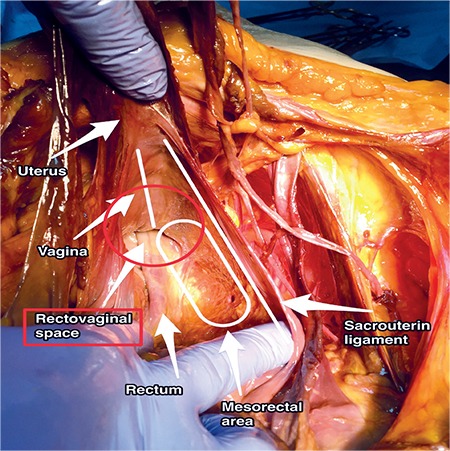
Rectovaginal space (cadaveric dissection)

The rectovaginal space is from the recto-uterine peritoneal fold (pouch of Douglas) to the level of the perineal body. Access to this area can be maintained by cutting the recto-uterine peritoneal structure between the insertions of the uterosacral ligament, which lie bilaterally. Its boundaries are (Figure 22):

- Anteriorly: Posterior wall of the vagina,

- Posteriorly: Anterior wall of the rectum,

- Laterally: Uterosacral ligament, rectal pillars.

### Clinical tip: Rectovaginal space

The recto-uterine, recto-vaginal pouch will be enclosed by severe adhesions due to endometriosis, tumor or abscess. Dissection of the vagina from the rectum and uterosacral ligaments after developing the pararectal space can maintain an extra field for surgery. The loose areolar tissue between the rectum and vagina can be bluntly dissected easily; however, the fatty tissue in this area belongs to the rectum. The rectal pillars are fibro-connective tissues, which are vascularized by the middle rectal arteries from the internal iliac artery over the cardinal ligament^(11)^.

### Clinical tip: Cervical fibroids

Both the vesicouterine and rectouterine pouch (anterior and posterior cul-de-sac) should be cut, the bladder should be dissected downward to the level of anterior vagina, and the ureter must be identified bilaterally. In particular, lateral to the rectovaginal space, the ureter is in close proximity to the uterosacral ligaments (at the lateral part of uterosacral ligaments) and meticulous dissection should be applied with regard to the vascular connections and depth of a cervical mass^(12)^.

## References

[1] Richard Drake, Wayne Vogl and Adam W M (2015.). Mitchell, Gray’s Anatomy for Students, 3rd Edition. Churchill Livingstone/Elsevier.

[2] Frank Netter (2014.). Atlas of Human Anatomy, 6th Edition. Saunders/ Elsevier.

[3] Murat Öz, Salim Erkaya, Bülent Özdal, Mehmet Mutlu Meydanlı, İlker Selçuk, Tayfun Güngör (2014). Retroperitoneal vasküler varyasyonlar ve jinekolojik onkoloji cerrahisinde önemi. Türk Jinekolojik Onkoloji Dergisi.

[4] Tuğba Tekelioğlu, Hasan Aykut Tuncer, Eda Adeviye Şahin, İlker Selçuk (2017.). Pelvisin Vasküler Anatomisi. In: Ali Ayhan, Hüsnü Çelik, Polat Dursun, editor. Jinekolog Onkolog Bakış Açısıyla; Postpartum Kanama. Ankara/Turkey. Güneş Tıp Kitabevleri.

[5] Selcuk I, Tatar I, Firat A, Gungor T, Huri E (2018.). Is corona mortis a historical myth? A perspective from gynecological oncologist. J Turk Ger Gynecol Assoc.

[6] Keith L Moore, Arthur F (2013.). Dalley and Anne M. R. Agur, Clinically Oriented Anatomy 7th Edition. Lippincott Williams & Wilkins.

[7] No authors listed (2016.). Susan Standring, Gray’s Anatomy, The Anatomical Basis of Clinical Practice, 41st Edition. Elsevier.

[8] Selcuk I, Yassa M, Tatar I, Huri E (2018). Anatomic structure of the internal iliac artery and its educative dissection for peripartum and pelvic hemorrhage. Turk J Obstet Gynecol.

[9] Michael S (2016.). Baggish and Mickey Karram, Atlas of Pelvic Anatomy and Gynecologic Surgery, 4th Edition. Elsevier.

[10] Fujii S, Takakura K, Matsumura N, Higuchi T, Yura S, Mandai M, et al (2007). Precise anatomy of the vesico-uterine ligament for radical hysterectomy. Gynecol Oncol.

[11] John T (2014.). Hansen, Netter’s Clinical Anatomy, 3rd Edition. Saunders/ Elsevier.

[12] Mustafa Sargon (2017.). Anatomi Akıl Notları, 2. Baskı. Ankara/Turkey. Güneş Tıp Kitabevleri.

